# Supplementary material for: Temporal Dynamics of Host Use by Drosophila suzukii in California’s San Joaquin Valley: Implications for Area-Wide Pest Management
Source: Insects. 2019 Jul 15;10(7):206. doi: 10.3390/insects10070206 (PMC6681292; doi:10.3390/insects10070206)
Supplement: Supplementary file 1 [file insects-10-00206-s001.pdf]

Table S1. Summary of monthly collections of various fruits from different locations in California's Central Valley. Fruits were collected during the ripening periods for each fruit species or cultivar. Most collections were at the Kearney Agricultural Research and Extension Center (Kearney Ag Center), where numerous fruits and cultivars are grown. The collected fruit were categorized as intact (no visible breaks in the skin) or damaged (fruits had obvious wounds, split, cracks, or overripen with partial skin weakening).

County	Location	Site	Day	Month	Year	Fruit	n	SWD	n	SWD
Fresno	Parlier, CA	Kearney Ag Center	26	Feb	2013	Mandarin	0	0	10	0
Fresno	Parlier, CA	Kearney Ag Center	18	May	2013	Cherry	51	11	.	.
Contra Costa	Brentwood, CA	Private farm	23	May	2013	Loquat	25	0	.	.
Fresno	Parlier, CA	Kearney Ag Center	25	May	2013	Cherry	90	180	.	
Fresno	Parlier, CA	Kearney Ag Center	31	May	2013	Cherry	33	26	75	97
Fresno	Parlier, CA	Kearney Ag Center	5	June	2013	Cherry	60	46	64	60
Contra Costa	Brentwood, CA	Private farm	7	June	2013	Loquat	25	0	23	5
Fresno	Parlier, CA	Kearney Ag Center	13	June	2013	Cherry	123	112	227	214
Fresno	Parlier, CA	Kearney Ag Center	15	June	2013	Nectarine	10	0	5	2
Fresno	Parlier, CA	Kearney Ag Center	25	June	2013	Peach	10	0	6	0
Fresno	Parlier, CA	Kearney Ag Center	30	June	2013	Nectarine	0	0	5	0
Fresno	Parlier, CA	Kearney Ag Center	2	July	2013	Peach	0	0	1	0
Fresno	Parlier, CA	Kearney Ag Center	13	July	2013	Peach	10	0	8	9
Fresno	Parlier, CA	Kearney Ag Center	19	July	2013	Peach	0	0	4	0
Fresno	Parlier, CA	Kearney Ag Center	25	July	2013	Nectarine	10	0	7	0
Fresno	Parlier, CA	Kearney Ag Center	25	July	2013	Plum	10	0	10	0
Fresno	Parlier, CA	Kearney Ag Center	27	July	2013	Peach	0	0	1	0
Fresno	Parlier, CA	Kearney Ag Center	7	Aug	2013	Plum	10	0	10	3
Fresno	Parlier, CA	Kearney Ag Center	26	Sept	2013	Apple	10	0	20	0
Fresno	Parlier, CA	Kearney Ag Center	26	Sept	2013	Fig	10	0	19	5
Contra Costa	Brentwood, CA	Small farm	26	Sept	2013	Pear	10	0	15	0
Fresno	Parlier, CA	Kearney Ag Center	26	Sept	2013	Plum	10	0	10	2
Fresno	Parlier, CA	Kearney Ag Center	26	Sept	2013	Pomegranate	0	0	10	0
Fresno	Parlier, CA	Kearney Ag Center	15	Oct	2013	Apple	10	0	21	0
Fresno	Parlier, CA	Kearney Ag Center	15	Oct	2013	Fig	10	0	20	0
Fresno	Parlier, CA	Kearney Ag Center	15	Oct	2013	Pomegranate	0	0	20	12
Fresno	Parlier, CA	Kearney Ag Center	25	Nov	2013	Apple	10	0	10	3
Fresno	Parlier, CA	Kearney Ag Center	25	Nov	2013	Fig	10	0	20	0

Table S1 cont'd

County	Location	Site	Day	Month	Year	Fruit	n	SWD	n	SWD
Fresno	Parlier, CA	Kearney Ag Center	25	Nov	2013	Persimmon	25	0	10	1
Fresno	Parlier, CA	Kearney Ag Center	25	Nov	2013	Mandarin	0	0	10	0
Fresno	Parlier, CA	Kearney Ag Center	25	Nov	2013	Navel orange	0	0	5	1
Fresno	Parlier, CA	Kearney Ag Center	25	Nov	2013	Pomegranate	0	0	20	0
Fresno	Parlier, CA	Kearney Ag Center	18	Dec	2013	Mandarin	0	0	15	0
Fresno	Parlier, CA	Kearney Ag Center	18	Dec	2013	Navel orange	0	0	5	0
Fresno	Parlier, CA	Kearney Ag Center	25	Mar	2014	Mandarin	0	0	10	0
Fresno	Parlier, CA	Kearney Ag Center	25	Mar	2014	Navel orange	0	0	5	0
Fresno	Parlier, CA	Kearney Ag Center	15	Apr	2014	Mandarin	0	0	10	2
Fresno	Parlier, CA	Kearney Ag Center	15	Apr	2014	Navel orange	0	0	5	0
Fresno	Parlier, CA	Kearney Ag Center	7	May	2014	Cherry	100	99	75	97
Fresno	Parlier, CA	Kearney Ag Center	14	May	2014	Cherry	59	57	.	.
Fresno	Parlier, CA	Kearney Ag Center	22	May	2014	Cherry	90	101	200	10
Fresno	Parlier, CA	Kearney Ag Center	23	May	2014	Navel orange	0	0	5	0
Fresno	Parlier, CA	Kearney Ag Center	9	June	2014	Cherry	0	0	118	5
Fresno	Parlier, CA	Kearney Ag Center	15	June	2014	Nectarine	15	0	13	0
Fresno	Parlier, CA	Kearney Ag Center	25	June	2014	Peach	15	0	11	2
Fresno	Parlier, CA	Kearney Ag Center	7	July	2014	Nectarine	15	0	10	0
Contra Costa	Brentwood, CA	Small farm	25	Jul	2014	Pear	10	0	5	103
Contra Costa	Brentwood, CA	Riparian area 2	25	Jul	2014	Wild plum	25	0	.	.
Fresno	Parlier, CA	Kearney Ag Center	26	July	2014	Plum	10	0	10	0
Fresno	Parlier, CA	Kearney Ag Center	26	July	2014	Peach	15	0	10	0
Contra Costa	Brentwood, CA	Riparian area 2	7	Aug	2014	Wild plum	25	0		
Fresno	Parlier, CA	Kearney Ag Center	25	Aug	2014	Plum	10	0	10	0
Contra Costa	Brentwood, CA	Small farm	26	Aug	2014	Pear	15	0		
Contra Costa	Brentwood, CA	Private farm	16	Sept	2014	Pear	15	0	3	1
Contra Costa	Brentwood, CA	Riparian area 1	16	Sept	2014	Cactus	30	0	20	1
Fresno	Parlier, CA	Kearney Ag Center	17	Sept	2014	Fig	10	0	20	110
Fresno	Parlier, CA	Kearney Ag Center	17	Sept	2014	Plum	0	0	10	0
Fresno	Parlier, CA	Kearney Ag Center	17	Sept	2014	Pomegranate	0	0	10	0
Fresno	Parlier, CA	Kearney Ag Center	25	Sept	2014	Raisin grapes	500	0	20	0
Fresno	Parlier, CA	Kearney Ag Center	26	Sept	2014	Apple	10	0	14	0

Table S1 cont'd

County	Location	Site	Day	Month	Year	Fruit	n	SWD	n	SWD
Fresno	Parlier, CA	Kearney Ag Center	5	Oct	2014	Apple	10	0	21	0
Fresno	Parlier, CA	Kearney Ag Center	5	Oct	2014	Fig	10	0	20	0
Fresno	Parlier, CA	Kearney Ag Center	5	Oct	2014	Raisin grapes	520	0	10	0
Fresno	Parlier, CA	Kearney Ag Center	5	Oct	2014	Pomegranate	0	0	20	0
Fresno	Parlier, CA	Kearney Ag Center	5	Nov	2014	Apple	0	0	20	1
Fresno	Parlier, CA	Kearney Ag Center	5	Nov	2014	Fig	0	0	20	0
Fresno	Parlier, CA	Kearney Ag Center	5	Nov	2014	Pomegranate	0	0	20	0
Fresno	Parlier, CA	Kearney Ag Center	18	Dec	2014	Persimmon	25	0	5	0
Fresno	Parlier, CA	Kearney Ag Center	18	May	2015	Cherry	50	0		
Fresno	Parlier, CA	Kearney Ag Center	25	May	2015	Cherry	100	0	235	2
Fresno	Parlier, CA	Kearney Ag Center	5	June	2015	Cherry	0	0	300	7
Fresno	Shaver Lake, CA	Sierra mountains	3	Oct	2016	Buckthorn	150	0	.	.
Fresno	Shaver Lake, CA	Sierra mountains	3	Oct	2016	Bitter berry	1000	0	.	.
Fresno	Shaver Lake, CA	Sierra mountains	17	Sept	2017	Bitter berry	3800	0	.	.
